# 

**Published:** 1898-08

**Authors:** 


### Communicated.

### LIST OF COMMITTEE'S FOR THE JOINT MEETING OF THE SOUTHERN BRANCH OF THE NATIONAL DENTAL ASSOCIATION AND THE LOUISIANA STATE DENTAL SOCIETY.

*To the Editor of " The American Journal of Dental Science."*

Sir:—By request of Dr. W. E. Walker, President of the Southern Branch National Dental Association, I submit to you for publication the following:

### NO. I. COMMITTEE ON ARRANGEMENTS, IN SUB-DIVISIONS AND TRANSPOTATION COMMITTEE.

Dr. Joseph Bauer, (S. B. N. D. A.,) Chairman, New Orleans, La.
Dr. Jules J. Sarrazin, (S. B. N. D. A.,) New Orleans, La.
Dr. C. L. Alexander, (S. B. N. D. A..) Charlotte, N. C.
Dr. Ed. M. Kettig, (S. B. N. D. A.,) Louisville, Ky.

### HOTELS AND QUARTERS COMMITTEE.

Dr. J. Rollo Knapp, (S. B. N. D. A.,) Chairman, New Orleans, La.
Dr. Joseph Bauer, (S. B. N. D. A.,) New Orleans, La.
Dr. Wallace Wood, Jr., (L. S. D. S.,) New Orleans, La.
Dr. L. D. Archinard, (L. S. D. S.,) New Orleans, La.

### HALL, EXHIBITS AND ARRANGEMENTS COMMITTEE.

Dr. L. D. Archinard, (L. S. D. S.,) Chairman, New Orleans, La.
Dr. Joseph Bauer, (S. B. N. D. A.,) New Orleans, La.
Dr. Phil. Friedrichs, (L. S. D. S.,) New Orleans, La.
Dr. Chas. Mermilliod, (L. S. D. S.,) New Orleans, La.
Dr. A. J. Foret. (L. S. D. S.,) New Orleans, La.

NO. 2. COMMITTEE ON CLINICS.

Dr. T. P. Hinman, (S. B. N. D. A.,) Chairman, Atlanta, Ga.
Dr. J. Rollo Knapp, (S. B. N. D. A.,) New Orleans, La.
Dr. R. K. Luckie, (S. B. N. D. A.,) Holly Springs, Miss.

COMMITTEE ON CLINICS—LOUISIANA STATE DENTAL SOCIETY.

Dr. And. G. Friedrichs, (L. S. D. S.,) Chairman, New Orleans, La.
Dr. J. F. Johnston (L. S. D. S.,) Ruston, La.
Dr. L. D. Archinard, (L. S. D. S.,) New Orleans, La.

NO. 3. COMMITTEE ON PUBLICATION.

Dr. Shep. W. Foster, (S. B. N. D. A.,) Chairman, ex-officio, Atlanta, Ga.
Dr. E. P. Beadles, (S. B. N. D. A.,) Danville, Va.
Dr. C. L. Alexander, (S. B. N. D. A.,) Charlotte, N. C.

NO. 4. COMMITTEE ON ORAL HYGIENE.

Dr. L. M. Cowardin, Chairman, Richmond, Va.
Dr. S. W. Foster, Atlanta, Ga.
Dr. J. Percy Corley, Greensboro, Ala.
Dr. I. Simpson, Rock Hill, S. C.
Dr. B. F. Arrington, Goldsboro, N. C.
Dr. J. N. Crouse, Chicago, Ill.
Dr. W. T. Arrington, Memphis, Tenn.
Dr. L Augspath, Little Rock, Ark.
Dr. S. Dickson, Bolivar, Tenn.
Dr. E. F. Adair, Harmony Grove, Ga.
Dr. F. J. S. Gorgas, Baltimore, Md.

NO. 5. COMMITTEE ON PROSTHETIC DENTISTRY.

Dr. J. A. Dale, Chairman, Nashville, Tenn.
Dr. Geo. Evans, New York, N. Y.
Dr. Ed. Eggleston, Richmond, Va.
Dr. Albert B. King, Baltimore, Md.
Dr. W. H. Morgan, Nashville, Tenn.
Dr. J. G. Fife, Dallas, Texas.
Dr. L. D. Carpenter, Atlanta, Ga.
Dr. W. H. Cook, Denton, Texas.
Dr. J. L. Wolf, Washington, D. C.
Dr. Jas. S. Knapp, New Orleans, La.

NO. 6. COMMITTEE ON ORTHODONTIA AND ORAL SURGERY.

Dr. Geo. Hardy, Chairman, Baltimore, Md.
Dr. L. M. Cowardin, Richmond, Va.
Dr. A. R. Melendy, Knoxville, Tenn.
Dr. J. E. Orrison, Baltimore, Md.
Dr. W. W. Corley, Talladega, Ala.
Dr. H. Marshall, Atlanta, Ga.
Dr. Jules J. Sarrazin, New Orleans, La.
Dr. E. P. Keerans, Charlotte, N. C.
Dr. R. C. Young, Anniston, Ala.
Dr. F. L. Wood, Roanoke, Va.
Dr. Wm. J. Younger, Chicago, Ill.

NO. 7. COMMITTEE ON OPERATIVE DENTISTRY.

Dr. J. G. Fife, Chairman, Dallas, Texas.
Dr. A. P. Johnston, Anderson, S. C.
Dr. J. Edwin Boozer, Baltimore, Md.
Dr. J. E. Orrison, Baltimore, Md.
Dr. B. Holly Smith, Baltimore, Md.
Dr. H. D. Boyd, Troy, Ala.
Dr. E. L. Hunter, Fayetteville, N. C.
Dr. L. A. Smith, Port Gibson, Miss.
Dr. Sam. Rambo, Montgomery, Ala.
Dr. C. Sill, New York, N. Y.
Dr. J. M. Quattlebaum, Columbia, S. C.
Dr. W. H. Burr, Madison, Ga.

NO. 8. COMMITTEE ON CHEMISTRY, METALLURGY AND ANATOMY.

Dr. Albert B. King, Chairman, Baltimore, Md.
Dr. J. H. Benton, New Berne, S. C.
Dr. M. C. Marshall, St. Louis, Mo.
Dr. Chas. S. Grindall, Baltimore, Md.
Dr. B. J. Quattlebaum, Winnsboro, S. C.
Dr. W. H. Ewald, Portsmouth, Va.
Dr. B. H. Teague, Aiken, S. C.

NO. 9. COMMITTEE ON PATHOLOGY, MATERIA MEDICA AND THERAPEUTICS.

Dr. H. H. Johnson, Chairman, Macon, Ga.
Dr. H. A. Parr, New York, N. Y.
Dr. R. H. Jones, Selma, N. C.
Dr. J. T. Calvert, Spartansburg, S. C.
Dr. E. B. Marshall, Rome, Ga.
Dr. R. K. Luckie, Holly Springs, Miss.
Dr. J W. Smith, Baltimore, Md.
Dr. F. Y. Clark, Saratoga, N. Y.
Dr. F. L. Wood, Roanoke, Va.
Dr. W. T. Coles, Newman, Ga.

NO. 10. COMMITTEE ON MICROSCOPY, HISTOLOGY AND BACTERIOLOGY.

Dr. W. T. Martin, Chairman, Yazoo City, Miss.
Dr. H. A. Lowrance, Athens, Ga.
Dr. S. J. Cockerill, Washington, D. C.
Dr. S. G. Holland, Atlanta, Ga.
Dr. F. C. Wilson, Savannah, Ga.
Dr. V. E. Turner, Raleigh, N. C.
Dr. W. R. Clifton, Waco, Texas.
Dr. B. Rutledge, Florence, S. C.
Dr. C. T. Brockett, Atlanta, Ga.
Dr. T. P. Hinman, Atlanta, Ga.
Dr. W. H. Ewald, Portsmouth, Va.
Dr. J. A. Tigner, Rome, Ga.

NO. 11. COMMITTEE ON APPLIANCES AND IMPROVEMENTS.

Dr. T. M. Allen, Chairman, Birmingham, Ala.
Dr. F. H. McAnnally, Jasper, Fla.
Dr. D. E. Everitt, Raleigh, N. C.
Dr. W. H. H. Thackston, Farmville, Va.
Dr. J. P. H. Brown, Augusta, Ga.
Dr. J. E. Orrison, Baltimore, Md.
Dr. J. Rollo Knapp, New Orleans, La.
Dr. Ed. D. Frost, Elizabeth, N. J.
Dr. Louis Dotterer, Charleston, S. C.

NO. 12. COMMITTEE ON EDUCATION, LITERATURE, AND VOLUNTARY AND SPECIAL ESSAYS.

Dr. T. P. Whitby, Chairman, Selma, Ala.
Dr. R. D. Seals, Ft. Smith, Ark.
Dr. J. A. Harris, Baltimore, Md.
Dr. J. A. Hall, Collinsville, Ala.
Dr. H. W. Morgan, Nashville, Tenn.
Dr. J. A. Chapple, Atlanta, Ga.
Dr. B. D. Brabson, Knoxville, Tenn.
Dr. R. A. McDonald, Griffin, Ga.
Dr. E. C. Kirk, Philadelphia, Pa.

ENTERTAINMENT COMMITTEE.

Dr. Joseph Bauer, (L. S. D. S.,) Chairman, New Orleans, La.
Dr. J. Rollo Knapp, (L. S. D. S.,) New Orleans, La.
Dr. C. V. Vignes, (L. S. D. S.,) New Orleans, La.
Dr. E. J. Zeidler, (L. S. D. S.,) New Orleans, La.

CARNIVAL BALLS INVITATIONS COMMITTEE.

Dr. J. Rollo Knapp, (S. B. N. D. A.,) Chairman, New Orleans, La.
Dr. Joseph Bauer, (S. B. N. D. A.,) New Orleans, Da.
Dr Wallace Wood, Jr., (L. S. D. S.,) New Orleans, La.
Dr. L. D. Archinard, (L. S. D. S.,) New Orleans, La.
Dr. E. Telle, (L. S. D. S.,) New Orleans, La.
Dr. V. K. Irion, (L. S. D. S.,) New Orleans, La.

C. L. Alexander, Corresponding Secretary. Southern Branch National Dental Association, Charlotte, N. C., July 12th, 1898.

### To Readers and Correspondents.

Communications intended for publication, and exchange journals and papers, should be addressed to the Editor of The American Journal of Dental Science, No. 845 N. Eutaw St. Baltimore, Md. All letters relating to the business of the Journal, or containing cash remittances, to be directed to Snowden & Cowman Mfg. Co., 9 W. Fayette Street, Baltimore, Md. Articles for publication should be received by the Editor at least two weeks previous to the first of the month on which the number in which it is designed for them to appear is issued.

☞ The American Journal of Dental Science will contain fifty pages of reading matter in each number, making a volume of about Six Hundred pages,

EXCLUSIVE OF ADVERTISEMENTS.

